# Heterogeneous immune cell composition in patients with combined immunodeficiency

**DOI:** 10.3389/fimmu.2026.1830231

**Published:** 2026-06-12

**Authors:** Jareb J. Pérez-Caraballo, Colleen M. Roark, Megan M. Dobrose, Ana Van den Rym, Aidé Tamara Staines-Boone, Yuridia Salazar-Galvez, Noemi Gomez-Hernandez, Jian Cui, Lauren E. Brown, Xin Zhen, Saul O. Lugo Reyes, Lizbeth Blancas-Galicia, Óscar de la Calle-Martín, Reem Mohammed, Rebeca Pérez de Diego, Rubén Martínez-Barricarte

**Affiliations:** 1 Division of Genetic Medicine and Clinical Pharmacology, Department of Medicine, Vanderbilt University Medical Center, Nashville, TN, United States; 2 Division of Molecular Pathogenesis, Department of Pathology, Microbiology and Immunology, Vanderbilt University Medical Center, Nashville, TN, United States; 3 Laboratory of Immunogenetics of Human Diseases, IdiPAZ Institute for Health Research, La Paz University Hospital, Madrid, Spain; 4 Immunology Service, Hospital de Especialidades Unidad Médica de Alta Especialidad (UMAE), 25 del Instituto Mexicano del Seguro Social (IMSS), Monterrey, Mexico; 5 Allergy and Clinical Immunology Service, Unidad Médica de Alta Especialidad, Centro Médico Nacional de Occidente IMSS, Guadalajara, Jalisco, Mexico; 6 Immune Deficiencies Laboratory, National Institute of Pediatrics, Health Secretariat, Mexico City, Mexico; 7 Immunology Department, Hospital de la Santa Creu i Sant Pau, Barcelona, Spain; 8 Section of Pediatric Allergy and Immunology, Department of Pediatrics, King Faisal Specialist Hospital and Research Centre, Riyadh, Saudi Arabia; 9 Vanderbilt Institute of Infection, Immunology and Inflammation, Vanderbilt University Medical Center, Nashville, TN, United States; 10 Vanderbilt Center for Immunobiology, Vanderbilt University Medical Center, Nashville, TN, United States; 11 Vanderbilt Genetics Institute, Vanderbilt University Medical Center, Nashville, TN, United States

**Keywords:** Arpc1b, Bcl10, combined immunodeficiency, cyTOF, ezrin, inborn errors of immunity, IRF4, mass cytometry

## Abstract

**Introduction:**

Combined immunodeficiencies (CIDs) are a severe class of inborn errors of immunity characterized by defective T cell development and function, often accompanied by impaired humoral and natural killer (NK) cell responses. Despite their shared clinical classification, the immunological heterogeneity within CIDs remains incompletely understood. This study aims to characterize the immune cell landscape in CID patients caused by disease-causing mutations in different genes to identify immunophenotypic patterns.

**Methods:**

We analyzed peripheral blood immune cells from four CID patients with disease-causing mutations in *ARPC1B, EZR, BCL10*, and *IRF4* using mass cytometry. Unbiased computational approaches were used to profile major immune populations and their subsets, and results were compared with healthy control samples to identify differences in immune cell frequencies and phenotypes.

**Results:**

All patients retained major immune populations, but their relative frequencies differed significantly from those of healthy controls. Patients with EZR and ARPC1B deficiency had markedly reduced CD4^+^ and CD8^+^ T cell frequencies, whereas the BCL10-deficient patient had a near absence of NK cells, highlighting mutation-specific immune distributions. Detailed T cell subset analyses revealed increased naïve and decreased memory T cells in patients with BCL10 and IRF4 deficiencies, indicative of impaired T cell activation and memory formation. In contrast, ARPC1B deficiency was associated with elevated memory T cells and reduced naïve cells, suggesting thymic output defects. The Ezrin-deficient patient maintained a naïve-to-memory T cell ratio similar to controls despite an overall reduction in T cells. B cell abnormalities were consistent across patients, characterized by increased naïve B cells, decreased memory B cells, and severely diminished plasmablast frequencies.

**Discussion:**

Our findings reveal pronounced immunological heterogeneity among CID patients caused by different genetic defects, challenging the notion that CIDs constitute a uniform entity. Disease-causing gene-specific alterations in immune cell composition and differentiation states underscore the complexity of CID pathophysiology. Comprehensive immunophenotypic profiling offers valuable insights into distinct mechanistic pathways and may guide the development of tailored therapeutic strategies to improve clinical outcomes for CID patients.

## Introduction

Inborn errors of immunity (IEIs) are a group of monogenic disorders that alter the development and/or function of the immune system. Clinically, they confer susceptibility to severe infection, autoimmunity, autoinflammation, allergy, or cancer, among others (1, 2). Combined immunodeficiencies (CIDs) are one of the most severe subgroups of IEIs. Unlike severe combined immunodeficiency (SCID), which typically presents in infancy with a near-complete absence of functional lymphocytes, CIDs are characterized by a quantitative or qualitative reduction in T lymphocytes and, often, also display impaired humoral immunity and NK functional defects (1, 3). Due to impaired adaptive immunity, these patients present with severe and recurrent life-threatening infections since childhood caused by a myriad of bacteria, fungi, viruses, and parasites (1, 4). Furthermore, some CIDs also present with syndromic features (such as failure to thrive or craniofacial features) and various degrees of immune dysregulation, including lymphoproliferation or autoimmunity (2, 5, 6). Early diagnosis and intervention are critical to improving outcomes, as untreated CIDs often result in significant morbidity and mortality.

With CID-causing genetic variants in over 100 genes, these diseases are mechanistically heterogeneous, providing a unique opportunity to learn basic mechanisms of T cell biology directly in humans (2). By studying patients with mutations that alter thymic function, TCR signaling, co-stimulatory pathways, or cytokine signaling, scientists have identified critical and non-redundant players in human T cell development and function (7–11). Furthermore, studies of CIDs have also shed light on the roles of cytoskeletal polymerization, apoptosis, DNA replication, and metabolism in the assembly of a proper T cell-mediated immune response by the study of mutations affecting these pathways (1, 12–14). Hence, the in-depth genetic, molecular, and immunological characterization of patients with CID offers a unique opportunity to learn basic mechanisms of human immunity. In this manuscript, we applied high-dimensional mass cytometry coupled with unbiased computational methods to examine immunological heterogeneity among patients with CID caused by disease-causing variants in four genes that affect distinct pathways.

## Materials and methods

### Patient population

We gathered samples and immunophenotyping data from 4 patients with CID caused by mutations in genes affecting different pathways to assess variability in immune cell compositions.

1. ARPC1B deficiency caused by the homozygous variant p.E300Gfs*7 (11, 14). ARPC1B is a subunit in the ARP2/3 complex, which acts as a template for the formation of new actin filaments, a process that plays a critical role in cell division, immunological synapse formation, and signal transduction in T cells (15, 16).
2. Ezrin deficiency caused by the homozygous mutation p.A127T (17). Ezrin is a key cytoskeletal protein and member of the ERM (ezrin-radixin-moesin) family that acts as a vital cross-linker between the plasma membrane and the actin cytoskeleton, allowing for a proper T cell activation and migration (18–20).
3. BCL10 (B-cell lymphoma/leukemia 10) deficiency caused by the homozygous mutation K63X (21). BCL10 is a member of the CBM complex (CARMA-BCL10-MALT1) critical for NF-κB signaling in immune cells (22).
4. IRF4 (Interferon regulatory factor 4) deficiency caused by the heterozygous mutation p.T95R (10). IRF4 is a critical transcription factor that regulates lymphocyte function and differentiation (23, 24).

A summary of the clinical presentation of these patients can be found in **Supplementary Table 1**.

### PBMC isolation

Peripheral Blood Mononuclear Cells (PBMCs) were isolated from whole blood using Ficoll-Hypaque density gradient centrifugation (Amersham-Pharmacia-Biotech, Buckinghamshire, UK). In addition to the patients mentioned above, we obtained samples from 23 healthy controls, aged 22 to 35 years. Although we tried to include age-matched controls, the lack of access to healthy pediatric donors limited our ability to establish fully representative normal ranges for the pediatric population. Consequently, our reference values may not entirely reflect age-specific immune profiles in children.

### Mass cytometry data acquisition and analysis

Data were either obtained from published reports or acquired using our Cytometry by time of flight (CyTOF) antibody panel (**Supplementary Table 2**) (17, 21, 25). Briefly, we stained 5 × 10^^6^ peripheral blood mononuclear cells (PBMCs) with Cisplatin-195Pt (Standard biotools, Boston, MA) to discriminate dead cells. We then blocked FcR using Human TruStain FcX (Biolegend, San Diego, CA), followed by staining with the antibody mix shown in **Supplementary Table 1**. After staining, we fixed the cells with paraformaldehyde and stained them with Cell-ID intercalator -Ir. We acquired the data from these stained PBMCs using a Helios 3 Mass Cytometer at the University of Maryland Mass Cytometry Core Laboratory. We aimed to acquire at least 100,000 cells per sample (**Supplementary Figure 2**). We exported the data as Flow Cytometry Standard (FCS) files and normalized them using EQ beads standards (Standard biotools) following the manufacturer’s instructions.

We then imported the FCS data into FlowJo 10.7.2 (Becton, Dickinson & Company, Ashland, OR). We removed dead cells, debris, and selected leukocytes based on CD45 and CD66b expression, following standard procedures (26). We then either performed classical biaxial gating as shown in **Supplementary Figure 1** or exported the pre-gated data from FlowJo and imported it into RStudio for multidimensional analysis. For the latter, we used the CATALYST package to arcsine-transform marker intensities with a cofactor of 5 and to perform subsequent analysis (27). We performed unsupervised clustering with FlowSOM and visualized the data with ggplot2. We used Prism 9 (GraphPad Software, San Diego, CA) to represent the frequencies obtained by manual gating.

## Results

### Altered immune cell distribution in CID patients

**Figure 1:**
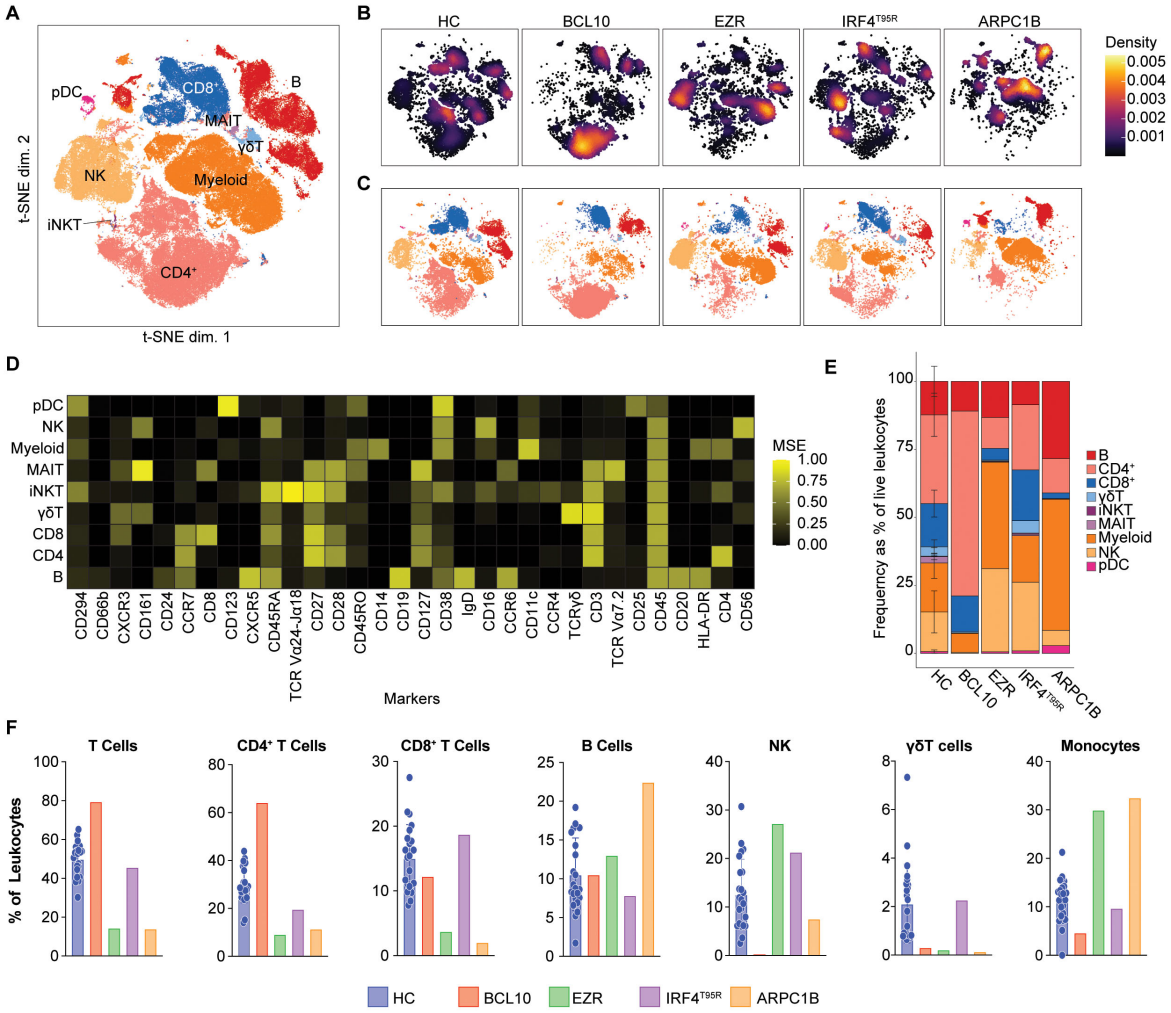
Overview of immune cell composition in patients with CID. **(A)** Dimensional reduction of CyTOF data using t-SNE. Each population is represented with a different color, and its name is shown. **(B)** t-SNE displaying density distribution and **(C)** population distribution of a representative healthy control (HC) and the four patients. **(D)** Heat map showing the intensities of the different markers in each population. **(E)** Stack bar graph showing the frequencies of the populations indicated in A as a percentage of total live leukocytes. **(F)** Graphs showing the biaxial gating results within the CD4^+^ T cell compartment.

To obtain an overview of the immune composition of patients compared with healthy controls, we performed an unbiased analysis that combined unsupervised clustering with manual clustering. For data visualization, we performed dimensional reduction by t-Distributed Stochastic Neighbor Embedding (t-SNE) (**Figures 1A–D**). We could identify 9 major immune cell populations (B, CD4^+^ T, CD8^+^ T, NK, pDC, Myeloid, MAIT, iNKT, γδT cells) and then studied the distribution between samples (**Figures 1A–C**). We observe that all these major populations were present in the four patients investigated; however, their distributions differed from those of the healthy controls, indicating altered frequencies of the subpopulations they contain (**Figures 1B, C**). We observed that T cell frequencies were low in Ezrin- and ARPC1B-deficient patients in both the CD4^+^ and CD8^+^ T cell compartments (**Figures 1E, F**). B cells were within normal ranges in all patients except the ARPC1B-deficient patient, who had elevated frequencies. NK cells were almost undetectable in patients with BCL10 deficiency, whereas the remaining patients had normal frequencies. Finally, the frequencies of myeloid cells and monocytes in Ezrin- and ARPC1B-deficient patients appear increased, as shown by the data presented as percentages of leukocytes (**Figure 1F**; **Table 1**). Overall, our data show distinct patterns of immune populations across all patients, without major lymphopenia. Given the differential distribution of patients compared with healthy controls, we analyzed each population separately (**Figures 1B, C**).

**Table 1:** Immunophenotyping frequencies obtained by biaxial gating.

Frequencies	HC range	BCL10	EZR	IRF4^T95R^	ARPC1B
As percentage of live leukocytes					
B	1.72 - 19.2	10.6	13.1	7.9	22.5 ↑
Switched B	0.29 - 2.88	0.026 ↓	0.37	0.099 ↓	0.014 ↓
Unswitched B	0.39 - 2.55	0.93	3.55 ↑	0.51	0.11 ↓
Naïve B	0.89-13.3	9.55	8.65	7.11	21.9 ↑
DN B	0.2 - 2.95	0.059 ↓	0.5	0.19 ↓	0.44
Plasmablasts (10E-3)	1.5-79	0 ↓	1.64	1.38 ↓	0 ↓
Transitional B	0.08 - 0.5	0.92	0.18	0.58 ↑	0.38
CD4^+^ T	14.1 - 43.9	64.4	9.36 ↓	19.8	11.6 ↓
T Regs	0.57 - 2.29	0.013 ↓	0.5 ↓	0.42 ↓	0.48 ↓
Follicular T	1.03 - 5.08	0.033 ↓	0.79 ↓	0.37 ↓	0.042 ↓
CD4^-^CD8^-^ T	0.45 - 1.48	0.98	0.29 ↓	3.22 ↑	0.12 ↓
CD4^+^CD8^+^ T	0.47 -1.80	0.75	0.086 ↓	0.42 ↓	0.03 ↓
CD4^+^ TEMRA	0.64 - 2.97	0.11 ↓	0.43 ↓	0.69	1.35
CD4^+^ Naïve	8.86 - 32.2	63.1	5.33 ↓	17.4	4.49 ↓
CD4^+^ Central Memory	1.26 - 5.99	0.83 ↓	1.71	0.47 ↓	3.71
CD4^+^ Effector Memory	0.41 - 2.46	0.082 ↓	0.45	0.34 ↓	1.49
CD8^+^ T	7.78 - 27.5	12.3	3.82 ↓	18.8	2.12 ↓
CD8^+^TEMRA	0.31 - 3.36	0.029 ↓	0.29 ↓	0.74	0.14 ↓
CD8^+^ Naïve	5.26 - 18.6	12.2	3.06 ↓	17.8	1.14 ↓
CD8^+^ Central Memory	0.27 - 1.66	0.041 ↓	0.32	0.091 ↓	0.66
CD8^+^ Effector Memory	0.081 - 0.59	0.004 ↓	0.1	0.093	0.17
Total NK	2.5 - 30.7	0.27 ↓	27.3	21.4	7.64
NK CD56^br^	0.06 - 0.41	0.019 ↓	0.46 ↑	0.039 ↓	0.18
NK CD56^dim^CD16^+^	1.53 - 21.50	0.14 ↓	22.5 ↑	20	3.17
NK CD56^dim^CD56^-^	0.16 - 10.8	0.1 ↓	4.12	1.28	4.24
Non-Classical Monocytes	1.10 - 7.95	0.54 ↓	1.65	1.91	6.66
Intermediate Monocytes	0.47 - 4.02	1.01	2.59	1.34	3.52
Classical Monocytes	2.21 - 13.40	3.17	25.8 ↑	6.54	22.4 ↑
pDC	0.12 - 0.84	0.4	1.06 ↑	0.46	0.49
mDC	1.5 - 11.4	1.42 ↓	4.29	6.43	15.2 ↑
γδ T Cells	0.64 - 7.33	0.33 ↓	0.24 ↓	2.29	0.16
iNKT	0.04 - 1.11	0.4	0.64	0.97	0.049
MAIT	0.02 - 1.73	0.013 ↓	0.039	0.031	0 ↓
As percentage of CD4^+^ T cells					
CD4^+^ TEMRA	1.46 - 7.87	0.18 ↓	4.64	3.46	11.6 ↑
CD4^+^ Naive	48.7 - 74.3	97.9	56.9	87.6 ↑	38.7
CD4^+^ CM	4.78 - 21.7	1.29 ↓	18.3	2.37 ↓	32 ↑
CD4^+^ EM	1.42 - 9.78	0.13 ↓	4.76	1.73	12.8 ↑
As percentage of CD8^+^ T cells					
CD8^+^ TEMRA	5.34 - 15.20	0.24 ↓	7.7	3.96 ↓	6.83
CD8^+^ Naive	63.7 - 84.9	99.2	80	94.7 ↑	53.9
CD8^+^ CM	1.23 - 12.8	0.33 ↓	8.32	0.48 ↓	31.1 ↑
CD8^+^ EM	0.36 - 5	0.035 ↓	2.64	0.5	8.06 ↑
As percentage of B cells					
Switched	4.44- 18.6	0.25 ↓	2.8 ↓	1.25 ↓	0.06 ↓
Unswitched	5.22 - 44.6	8.77	27.2	6.44	0.48 ↓
Naive	34.5 - 82.8	90.4 ↑	66.2	89.9 ↑	97.5 ↑
DN	2.28 - 17.9	0.56 ↓	3.83	2.41	1.95 ↓
Plasmablasts	0.14 - 0.48	0 ↓	0.013 ↓	0.018 ↓	0 ↓
Transitional B	0.57 - 5.45	8.74 ↑	1.4	7.31 ↑	1.71

Red indicates reduced frequencies, while green indicates increased frequencies. Arrow pointing up means higher in the patient than in healthy controls. Arrow pointing down means to opposite.

### CID patients exhibit diverse T cell phenotypes

**Figure 2:**
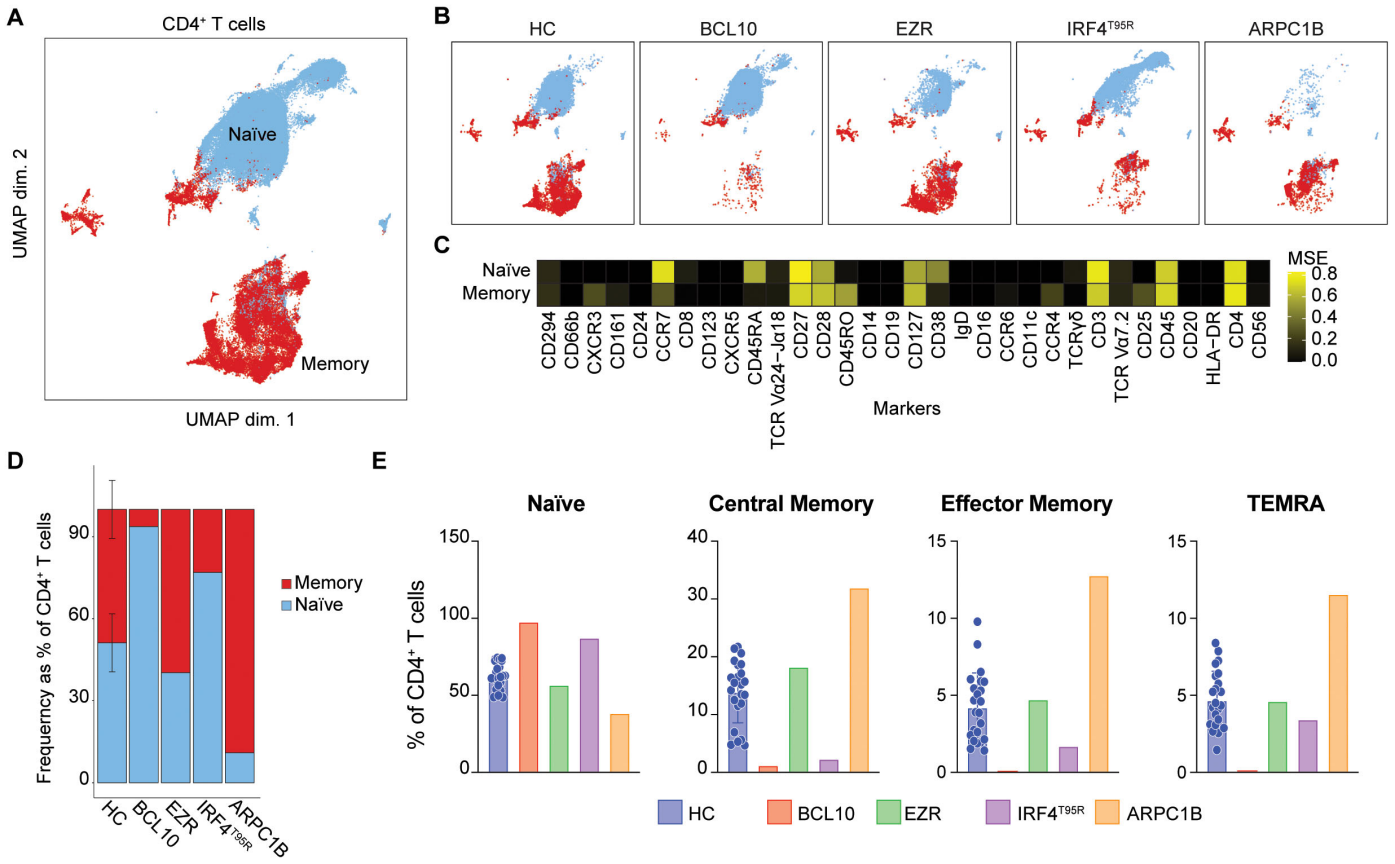
CD4^+^ T cell immunophenotyping. **(A)** Dimensionality reduction of CD4^+^ T cell subclusters using UMAP, based on data from **Figure 1**. Naïve and Memory populations are labeled in the figure. **(B)** UMAP from A separated by condition. **(C)** Heat map showing marker intensity for the two populations highlighted in A. **(D)** Stacked bar graph depicting the frequencies of Naïve and Memory cells across different conditions as a percentage of total CD4^+^ T cells. **(E)** Graphs illustrating biaxial gating results within the CD4^+^ T cell compartment.

**Figure 3:**
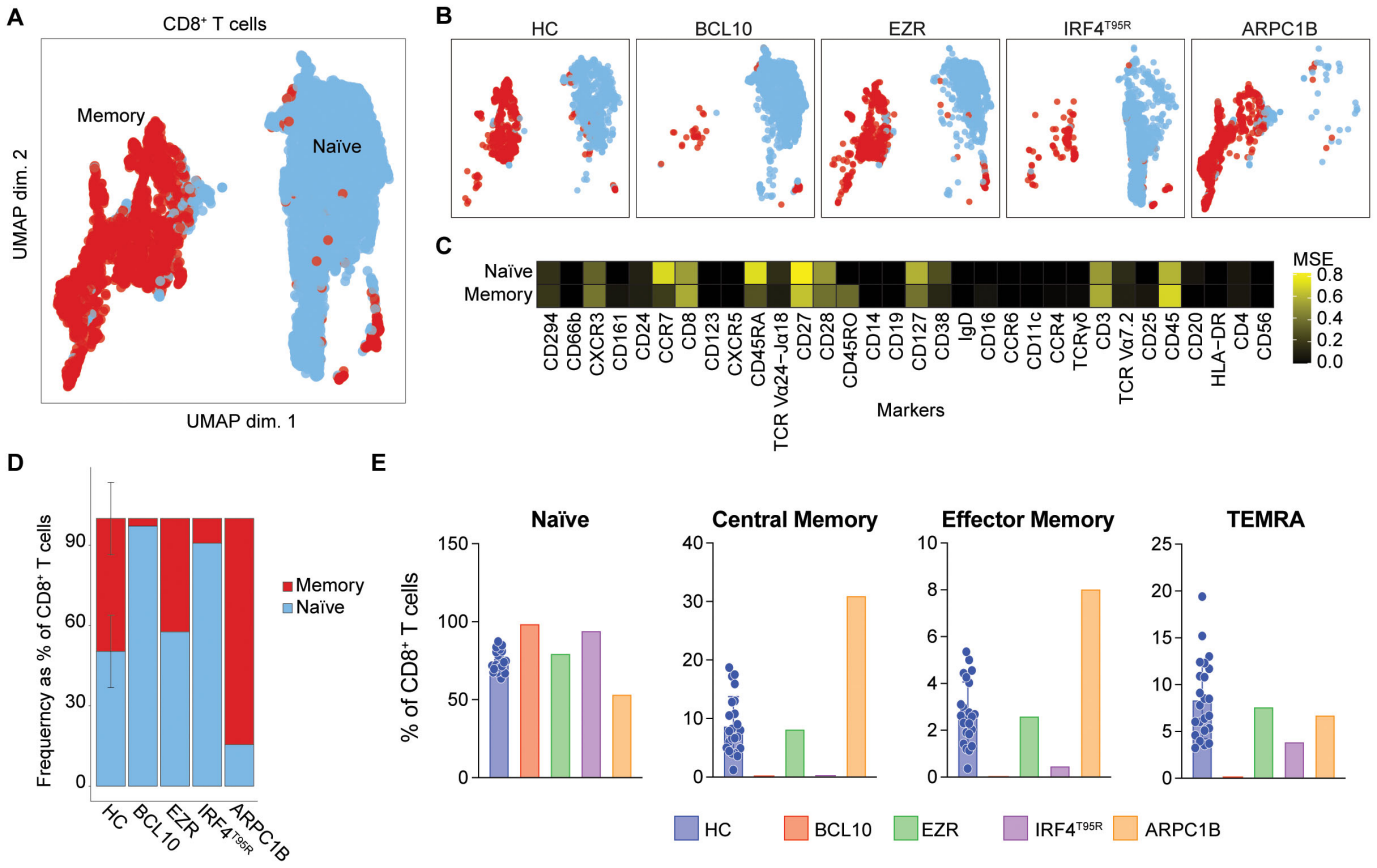
CD8^+^ T cell immunophenotyping. **(A)** Dimensional reduction using UMAP of CD8^+^ T cell subclusters from **Figure 1**. The main populations, Naïve and Memory, are indicated in the figure. **(B)** UMAP from A separated by condition. **(C)** Heat map displaying marker intensity for the Naïve and Memory populations. **(D)** Stacked bar graph showing the frequencies of the populations in A across different conditions as a percentage of CD8+ T cells. **(E)** Graphs illustrating biaxial gating results within the CD8^+^ T cell compartment.

In the previous section, we observed an aberrant spatial distribution within the CD4^+^ and CD8^+^ T cell compartments. Hence, we subset the data for these populations and analyzed them independently to achieve better granularity. Using unsupervised clustering, we found two major populations in the CD4^+^ and CD8^+^ T cell compartments, corresponding to Naïve and Memory T cells (**Figures 2**, **3**). We observed that in both compartments, BCL10-deficient and IRF4^T95R^ patients showed an increased frequency of naïve T cells and a reduced frequency of memory T cells, suggesting defective T cell activation (**Figures 2A–D**, **3A–D**). Interestingly, the ARPC1B-deficient patient shows the opposite pattern, with increased memory and reduced naïve cells, which may be secondary to a thymic defect (**Figures 2A–D**, **3A–D**). Despite a lower total T cell count in the ezrin-deficient patient, the proportions of naïve vs memory T cells within the corresponding T cell compartments were comparable to those in healthy controls (**Figures 2A–D**, **3A–D**; **Supplementary Figure 3**). When we studied the percentages of naïve, central memory, effector memory and TEMRA within the CD4^+^ and CD8^+^ T cell compartments we saw no subpopulation driving the phenotypes described above indicating that all the memory T cells are affected to the same extent except the CD8^+^ TEMRA population which is normal contrary to total memory, central memory and effector memory that are increased in the ARPC1B deficient patient (**Figures 2**, **3**; **Supplementary Figure 3**; **Table 1**). Our data show that, despite CIDs altering T cell development, activation, and/or function, the nature and extent of the defect vary with the genetic etiology and the affected pathway.

### The B cell compartment is similarly impacted in our CID patients

**Figure 4:**
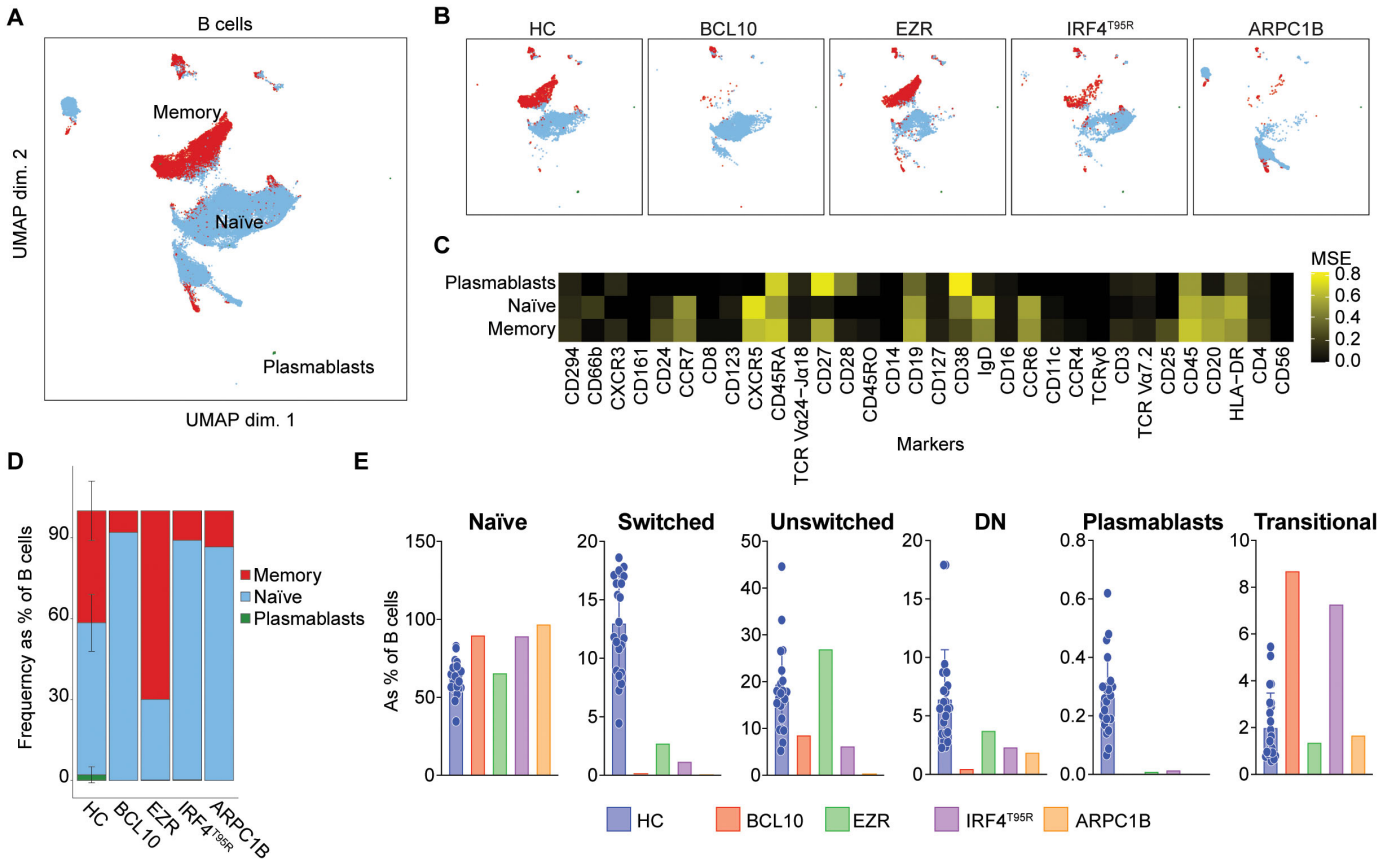
B cell immunophenotyping. **(A)** UMAP of the B cell subcluster from **Figure 1**, with the three main populations (Naïve, Memory, and Plasmablasts) indicated. **(B)** UMAP from A separated by condition. **(C)** Heat map showing marker intensity for the three populations highlighted in A. **(D)** Stacked bar graph displaying the frequencies of these populations across different conditions as a percentage of total B cells. **(E)** Bar graphs illustrating the biaxial gating results within the B cell compartment.

In contrast to the divergent T cell phenotypes described above, analysis of the B cell compartment revealed a strikingly more uniform picture across all four patients. B-cell activation or development is often altered in CIDs (2). A limitation in the study of B cell phenotypes is the difficulty in discerning whether a potential defect is intrinsic to B cells, caused by the CID-causing mutations, or extrinsic to B cells due to T cell dysfunction. To better understand the B cell phenotype in our patients, we subseted the B cell data and analyzed it in isolation to obtain a more detailed phenotypic characterization. Contrary to the phenotypes observed in T cells, B cell phenotypes are more homogenous among these 4 patients (**Figures 4A–D**). All but the Ezrin-deficient patient showed higher naïve and lower memory B cells consistent with impaired B cell activation (**Figures 4A–D**). Along those lines, all patients have lower frequencies of switched B cells, indicating impaired class switching due to these mutations (**Figure 4E**). Except in Ezrin-deficient patients, unswitched B cells fall within the lower range observed in controls. Interestingly, these four patients show severely reduced plasmablast frequencies, which explain their low antibody titers (**Figure 4E**; **Table 1**; **Supplementary Table 3**). Overall, although we don’t fully understand the origin of the B cell defects observed in this group of CID patients, our data show a homogeneous impairment of B cell activation, class switching, and plasmablast differentiation, leading to a reduction in at least one immunoglobulin isotype.

## Discussion

The primary objective of this study was to perform a comprehensive immune profiling of patients with CIDs caused by mutations in different pathways. We selected CyTOF for this purpose because it enables simultaneous measurement of over 40 markers per cell, providing a level of detail and breadth that conventional flow cytometry cannot match due to its limited multiplexing capacity (28). While spectral flow cytometry can accommodate panel sizes comparable to those of mass cytometry (29), CyTOF’s use of metal isotope tags significantly reduces spectral overlap and autofluorescence, resulting in clearer and more accurate data. Additionally, CyTOF data requires minimal to no compensation and exhibits very low batch effects, facilitating automated computational analysis of samples processed on different days (30). Although CyTOF has lower throughput and higher costs than conventional and spectral flow cytometry, its superior ability to analyze complex cellular phenotypes and reduced batch-to-batch variability made it the optimal choice for this study. Moreover, given the low number of samples of this study, driven by the rarity of CIDs, CyTOF’s limited throughput did not pose a significant limitation for our analysis. Using this method, our study revealed pronounced alterations in immune cell distribution compared with healthy controls, highlighting the heterogeneity of immune dysregulation across different CIDs. Using unbiased clustering and dimensionality reduction analyses, we identified all major immune populations in the patients studied, showing that there is no complete lymphopenia in this group (**Figure 1**). However, we observed alterations in their relative frequencies depending on the disease-causing mutations. Notably, patients with Ezrin and ARPC1B deficiencies exhibited significantly reduced frequencies of CD4^+^ and CD8^+^ T cells, whereas the BCL10-deficient patient displayed a near-absence of NK cells. These findings collectively show that the distribution of immune subsets in CID patients is distinctly altered in a mutation-specific manner. Such differences suggest that genetic factors influence not only the presence but also the proportional representation of immune cells, potentially affecting immune competence and susceptibility to infections.

Delving deeper into adaptive immunity, our subset analyses of T and B lymphocytes revealed divergent patterns of dysfunction associated with specific genetic mutations (**Figures 2**–**4**). In the T cell compartment, deficiencies in BCL10 and IRF4^T95R^ were characterized by increased naïve T cell frequencies and concomitant reductions in memory subsets in both CD4^+^ and CD8^+^ populations, suggesting impaired T cell activation or defective memory formation. In contrast, the ARPC1B-deficient patient exhibited an inverse pattern, with elevated memory T cells and diminished naïve T cells, potentially reflecting impaired thymic output or altered peripheral differentiation. Interestingly, the Ezrin-deficient patient, despite an overall reduction in T cell numbers, maintained a naïve-to-memory ratio comparable to healthy controls, indicating a distinct pathogenic mechanism (**Figures 2**, **3**). In contrast to the heterogeneous T cell phenotypes, B cell alterations were more consistent across the cohort, with most patients showing increased naïve B cells, decreased memory B cells, and a marked reduction in plasmablast frequencies indicative of defective class switching and impaired terminal differentiation. These B cell defects may arise from intrinsic B cell dysfunction or secondary effects of T cell abnormalities, highlighting the complex interplay between lymphocyte subsets in CID pathogenesis (**Figure 4**). Regardless of the origin of this B cell defect, the ultimate consequence is reduced levels of at least one immunoglobulin isotype in all patients (**Supplementary Table 3**). Together, our data reveal that while CID mutations variably disrupt T cell development and activation, they converge on impairing humoral immunity, underscoring the importance of dissecting both intrinsic and extrinsic mechanisms to inform targeted interventions.

We previously performed extensive immunophenotyping on patients with EZR, IRF4, BCL10, and ARPC1B deficiencies (10, 11, 17, 21). Hence, although individual data are not the most novel aspect of this work, our study’s innovation lies in the comparative analysis of more than 30 immune cell populations in CID patients using uniform CyTOF panels and advanced computational methods. This multidimensional approach provided new insights beyond the traditional T cell–focused CID research. We acknowledge that our analysis is limited to a group of patients representing only a small fraction of the genetic etiologies of CID, and this study should be repeated with a larger cohort to obtain a complete understanding of the variability in immune population frequencies caused by this disease. Furthermore, another limitation of our study is the inability to obtain absolute blood counts, as blood samples experienced variable transit times before reaching our laboratory. This variability hindered our capacity to directly compare absolute counts between samples. Therefore, we chose to perform our analysis on PBMCs by examining cell frequencies instead. Yet our data show substantial heterogeneity among patients diagnosed with CID, raising a broader conceptual question about the diagnostic category itself. CID is currently defined as a T cell defect substantial enough to impair adaptive immunity, yet insufficient to cause the complete lymphopenia that characterizes SCID (8). What our data show is that this shared definition conceals mechanistically different pathologies: impaired activation (BCL10, IRF4), presumed thymic output (ARPC1B), and T cell lymphopenia with preserved subset architecture (Ezrin) represent distinct cellular phenotypes that converge on a similar clinical disease. While the CID label is valuable as a *triage* category signaling disease severity and treatment urgency, it is insufficient as a mechanistic framework. Deep immunophenotyping, as performed here, will allow for an individualized understanding that can inform targeted cellular or molecular therapies and aligns with the broader movement in the field toward mutation-defined diagnostic nomenclature.

## Data Availability

The raw data supporting the conclusions of this article will be made available by the authors, without undue reservation.
