# Supplementary material for: Heterogeneous immune cell composition in patients with combined immunodeficiency
Source: Front Immunol. 2026 Jun 12;17:1830231. doi: 10.3389/fimmu.2026.1830231 (PMC13303123; doi:10.3389/fimmu.2026.1830231)
Supplement: Supplementary file 1 [file DataSheet1.docx]

**SUPPLEMENTARY MATERIAL**

**
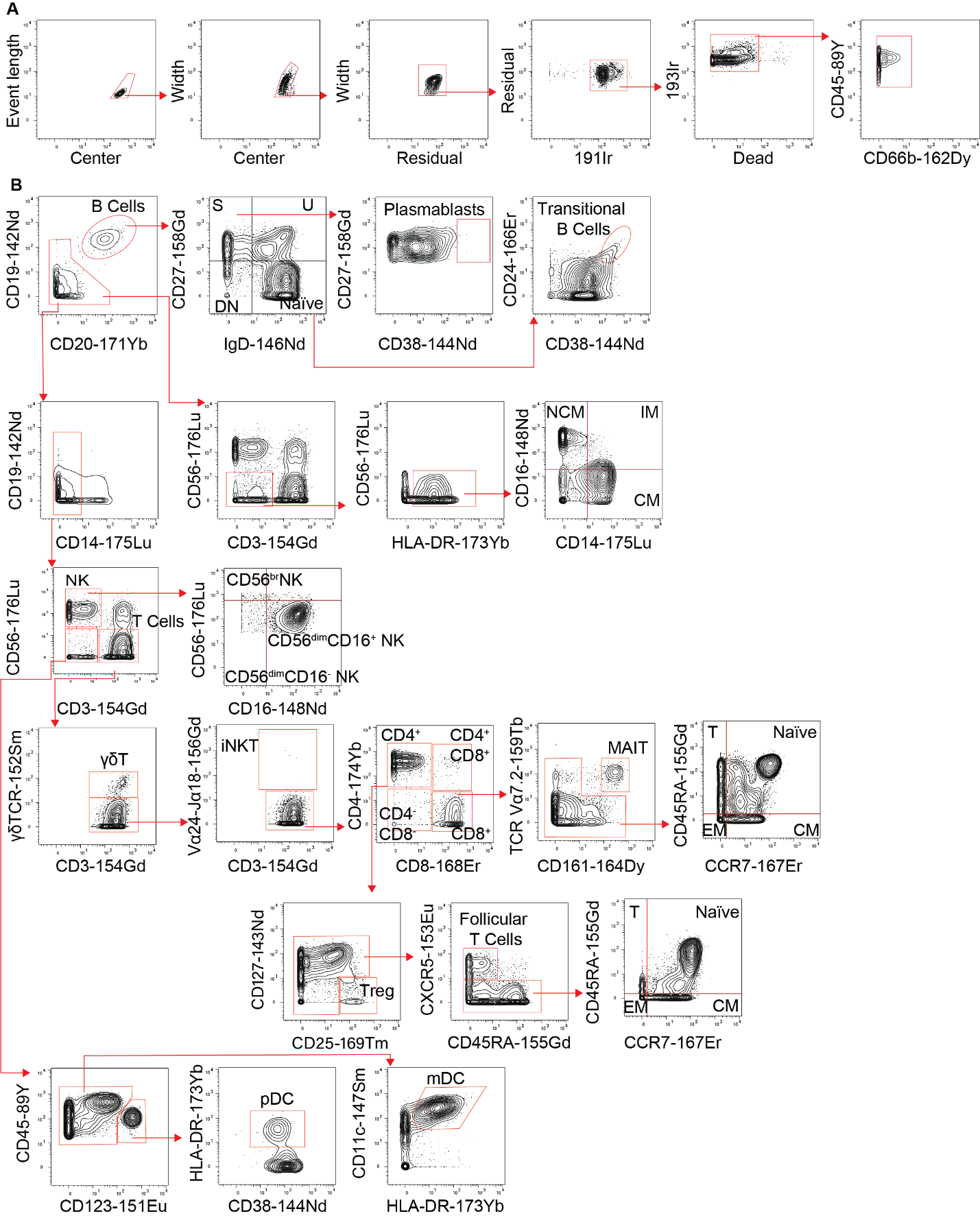
**

**Supplementary figure 1: biaxial gating example. A)** pregating before import into R. **B)** Manual gating strategy to obtain immune population frequencies**.**

**
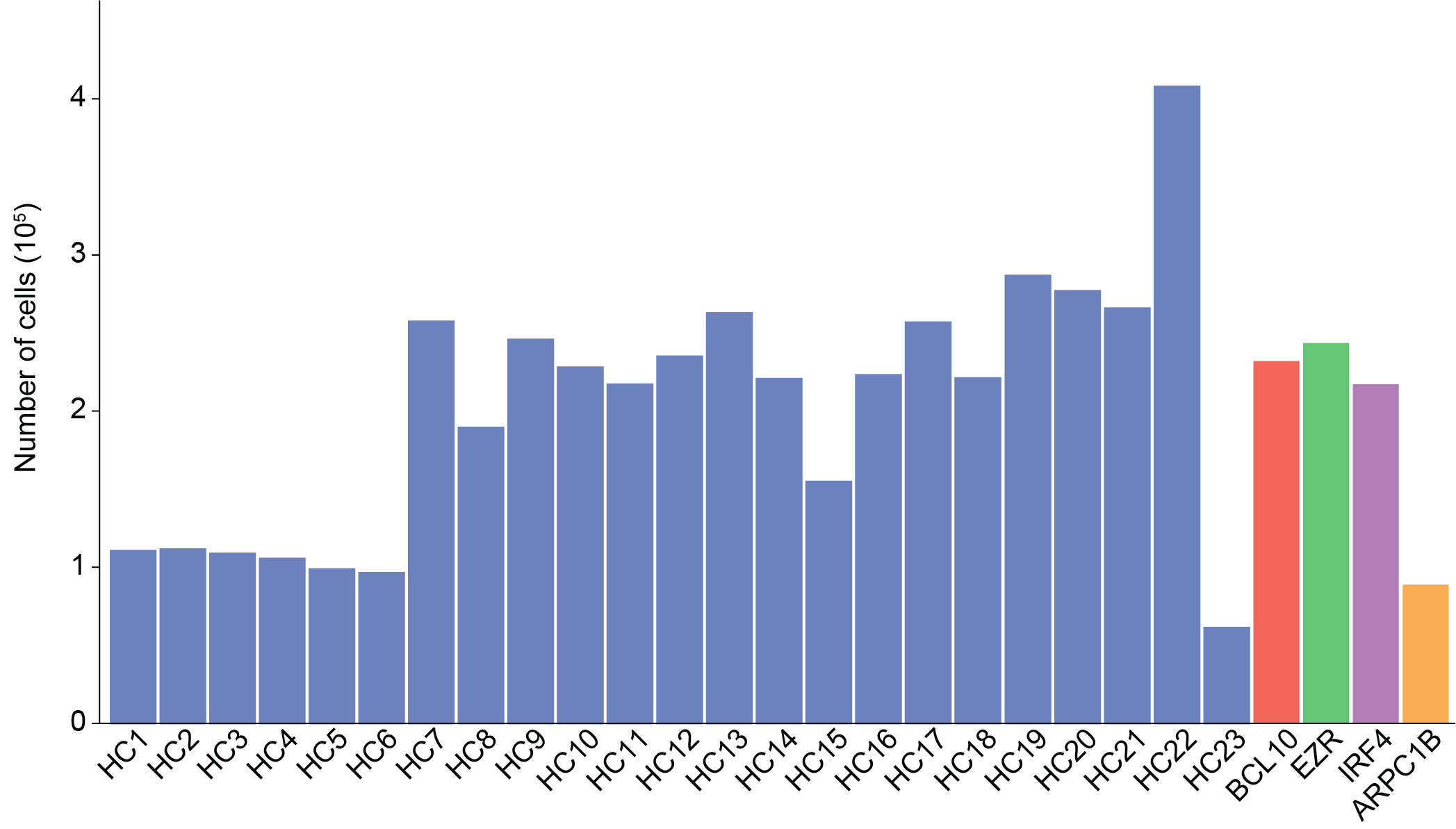
**

**Supplementary figure 2: quantification of the number of cells analyzed per sample after the pregating step shown in Fig. S1A.**

**
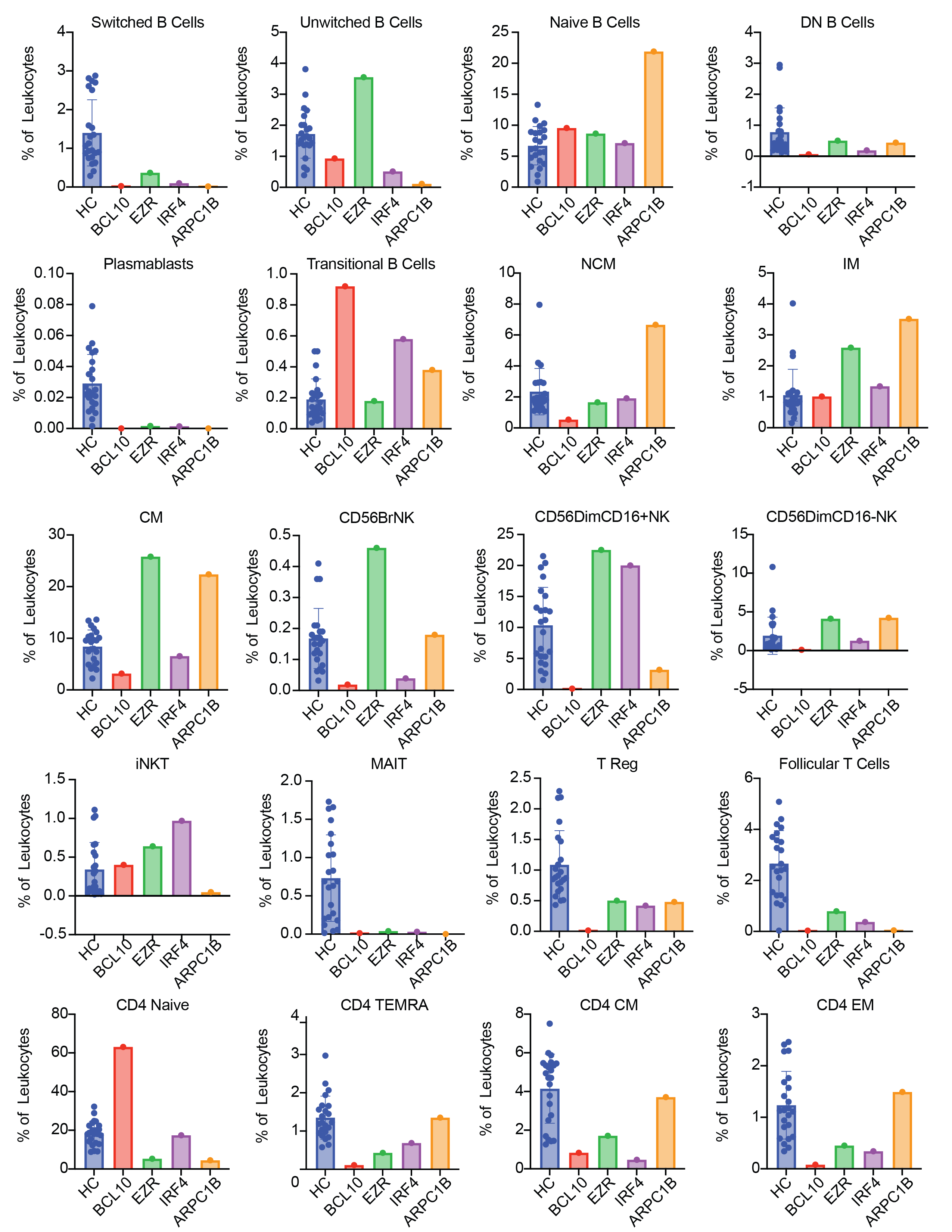
**

**
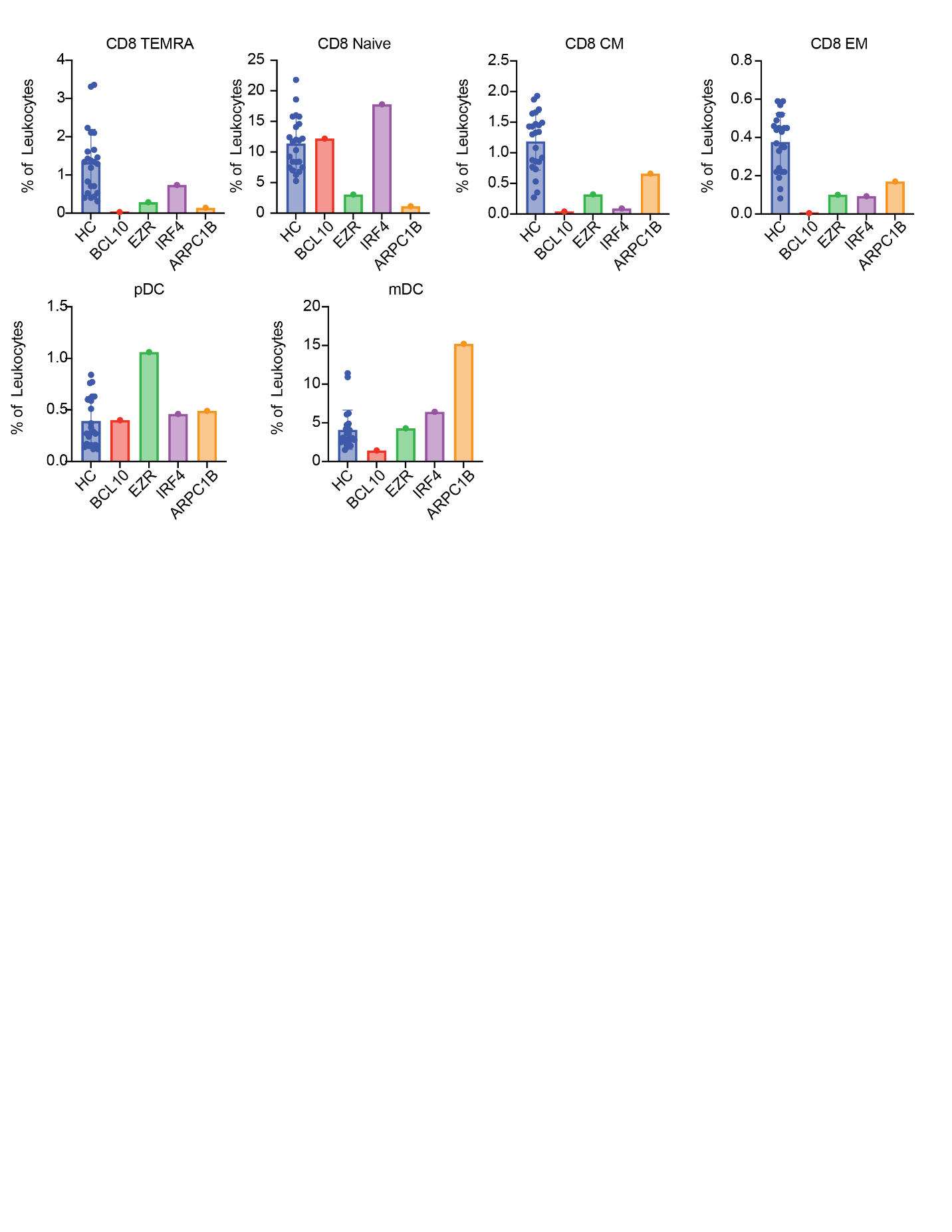
**

**Supplementary figure 3: Additional immunophenotyping graphs.**

**Supplementary table 1: Clinical summary**

| Gene | Mutation | Zygosity | Consanguineous | Family history | Age of diagnosis | Gender | Main Symptoms | Alive | Treatment | Age at CyTOF | Ref |  |
| --- | --- | --- | --- | --- | --- | --- | --- | --- | --- | --- | --- | --- |
| *BCL10* | K63X | Hom | Yes | Yes | Newborn | Female | Bacterial pneumonia | Yes | Prophylactic antibiotic  IVIG  HSCT | 6mo | 1 |  |
| *EZR* | A129T | Hom | Yes | No | 28 yo | Female | Candidiasis  Pinworms  Eczema  *Giardia lamblia* infection | Yes | Prophylactic antibiotic | 32yo | 2 |  |
| *ARPC1B* | E300Gfs*7 | Hom | Yes | Yes | 1 mo | Male | Eczema and other skin lesions  Recurrent bacterial and fungal respiratory infections  Bronchiolitis obliterans | No | Prophylactic antibiotic  Immunosuppressants  IVIG  Subcutaneous IFN-β | 33yo | 3 |  |
| *IRF4* | T95R | Het | No | No | 6 mo | Male | PJP  Recurrent respiratory infections  Viral Diarrhea  BCGitis | Yes | Prophylactic antibiotic  Prophylactic antifungals  IgG replacement  HSCT | 3yo | 4 |  |

PJP: *Pneumocystis jirovecii* pneumonia

IVIG: Intravenous immunoglobulin

HSCT: Hematopoietic stem cell transplantation

mo: Months old

yo: Years old

| **Antibody** | **Clone** | **Company** | **Isotope** | **Dilution** |
| --- | --- | --- | --- | --- |
| Anti-Human CD45 | H130 | Standard BioTools | 89Y | 1:50 |
| Anti-Human CD19 | HIB19 | Standard BioTools | 142Nd | 1:50 |
| Anti-Human 127/IL7Ra | A019D5 | Standard BioTools | 143Nd | 1:50 |
| Anti-Human CD38 | HIT2 | Standard BioTools | 144Nd | 1:50 |
| Anti-Human IgD | IA6-2 | Standard BioTools | 146Nd | 1:50 |
| Anti-Human CD11c | Bu15 | Standard BioTools | 147Sm | 1:50 |
| Anti-Human CD16 | 3G8 | Standard BioTools | 148Nd | 1:50 |
| Anti-Human CD194/CCR4 | L291H4 | Standard BioTools | 149Sm | 1:50 |
| Anti-Human CD123/IL-3R | 6H6 | Standard BioTools | 151Eu | 1:50 |
| Anti-Human TCRgd | 11F2 | Standard BioTools | 152Sm | 1:50 |
| Anti-Human CD185/CXCR5 | RF8B2 | Standard BioTools | 153Eu | 1:50 |
| Anti-Human CD3 | UCHT1 | Standard BioTools | 154Sm | 1:50 |
| Anti-Human CD45RA | HI100 | Standard BioTools | 155Gd | 1:50 |
| Anti-Human CD27 | L128 | Standard BioTools | 158Gd | 1:50 |
| Anti-Human CD28 | CD28.2 | Standard BioTools | 160Gd | 1:50 |
| Anti-Human CD66b | 80H3 | Standard BioTools | 162Dy | 1:50 |
| Anti-Human CD183/CXCR3 | G025H7 | Standard BioTools | 163Dy | 1:50 |
| Anti-Human CD161 | HP-3G10 | Standard BioTools | 164Dy | 1:50 |
| Anti-Human CD45RO | UCHL1 | Standard BioTools | 165Ho | 1:50 |
| Anti-Human CD24 | ML5 | Standard BioTools | 166Er | 1:50 |
| Anti-Human CD197/CCR7 | G043H7 | Standard BioTools | 167Er | 1:50 |
| Anti-Human CD8 | SK1 | Standard BioTools | 168Er | 1:50 |
| Anti-Human CD25 | 2A3 | Standard BioTools | 169Tm | 1:50 |
| Anti-Human CD20 | 2H7 | Standard BioTools | 171Yb | 1:50 |
| Anti-Human HLA-DR | L243 | Standard BioTools | 173Yb | 1:50 |
| Anti-Human CD4 | SK3 | Standard BioTools | 174Yb | 1:50 |
| Anti-Human CD56 | NCAM16.2 | Standard BioTools | 176Yb | 1:50 |
| Anti-Human CD196/CCR6 | G034E3 | Standard BioTools | 141Pr | 1:25 |
| Anti-Human CD14 | M5E2 | Standard BioTools | 175Lu | 1:25 |
| Anti-Human TCR Vα24/Jα18 | 6B11 | Biolegend | 156Gb | 1:25 |
| Anti-Human TCR Vα7.2 | 3C10 | Biolegend | 159Tb | 1:25 |
| Anti-Human CD294 | BM16 | Biolegend | 161Dy | 1:25 |

**Supplementary table 2: Antibody panel**

**Supplementary table 3: Immunoglobulin levels (g/l)**

| Ig Isotype | BCL10 | EZR* | ARPC1B | IRF4 | Ref. values |
| --- | --- | --- | --- | --- | --- |
| IgG | 6 | 3.91 | 1.87 | 1.69 | 6.2-16.85 |
| IgA | <0.5 | 0.218 | 9.68 | 0.06 | 0.5-3.82 |
| IgM | <0.25 | 0.268 | 4.2 | 0.09 | 0.4-2.3 |

* The Ezrin-deficient patient has progressive B cell loss that can be reflected by a gradual reduction of IG levels.

**References**

1. Solis, B. G. *et al.* Clinical and Immunological Features of Human BCL10 Deficiency. in (2021) doi:10.21203/RS.3.RS-807424/V1.

2. García-Solís, B. *et al.* Inherited human ezrin deficiency impairs adaptive immunity. in *J. Allergy Clin. Immunol.* 152, 997-1009.e11 (2023).

3. Vásquez-Echeverri, E. *et al.* Is Your Kid Actin Out? A Series of Six Patients With Inherited Actin-Related Protein 2/3 Complex Subunit 1B Deficiency and Review of the Literature. in *J. Allergy Clin. Immunol. Pract.* 11, 1261-1280.e8 (2023).

4. IRF4 International Consortium *et al.* A multimorphic mutation in IRF4 causes human autosomal dominant combined immunodeficiency. in *Sci. Immunol.* 8, eade7953 (2023).
